# Modeling Neglected and Underutilized Crops for Future Food Resilience: A Regional MaxEnt Workflow

**DOI:** 10.1002/pei3.70115

**Published:** 2026-02-26

**Authors:** Daniel J. Winstead, Michael G. Jacobson

**Affiliations:** ^1^ Department of Ecosystem Science and Management, College of Agriculture Science The Pennsylvania State University University Park Pennsylvania USA

**Keywords:** agrobiodiversity, crop suitability, food resilience, MaxEnt, neglected and underutilized crops (NUS)

## Abstract

Increasing agrobiodiversity is a widely supported idea and prevalent topic in academic discussion recently as a means to combat the effects of climate change. However, there is a lack of connection between academic discussion and application. Our study aims to create a high‐throughput MaxEnt workflow design that can be used to predict which neglected and underutilized crops (NUS) to use in any regional area with limited occurrence data. Our study reveals possible candidates for highly resilient NUS and that geospatial effects of climate change on these NUS were not homogenous. This tool can be deployed to help smallholders decide the most appropriate NUS to develop in any area based on crop suitability of current and future conditions.

## 1 Introduction

As global changes in population and climate increase, our current agricultural systems must adapt to produce enough reliable food for the entire population. The consensus among food demand projections is that there will need to be an increase in food production upwards of 50% by 2050 to account for increased global population (van Dijk et al. 2021). This issue compounds with increasing temperatures and more persistent drought in many tropical and subtropical areas (Winstead and Jacobson 2024). The projected changes in climate and population warrant actionable steps for greater food resilience. Increasing agrobiodiversity and using tools for the selection of appropriate climate‐tolerant crops are relevant and acknowledged solution strategies (Mabhaudhi et al. 2017; Mugiyo et al. 2021; Rosero et al. 2020; Winstead et al. 2023; Winstead and Jacobson 2022, 2024). Currently, 83% of calories consumed in the world are from only 10 different crops (Tilman et al. 2011). Increasing agrobiodiversity and appropriate cropping methods is not only a practice that cares for soil health and future crop production, but it also increases sustainable livelihoods for farmers. Large‐scale monocropping and many current agronomic systems usually do not promote soil health or the ability to cope with natural disasters (flood and drought), disease, and long‐term climate change (Belete and Yadete 2023; National Academies of Sciences 2024; Renwick et al. 2021).

Increasing the use of neglected and underutilized crops (NUS), although an increasingly talked about topic in academia, has had little actionable support on the ground (Mabhaudhi et al. 2017). This is despite the fact that the inclusion of NUS into cropping systems has had considerable academic support due to their desirable qualities in nutrition, climate resilience, disease resistance, cultural importance, and economic potential (Chivenge et al. 2015; Mabhaudhi et al. 2017; Winstead et al. 2023; Winstead and Jacobson 2024). Neglected and underutilized crops have been scaled up in the past with success, such as quinoa as a cash crop. This is especially successful when using a more holistic *conservation‐production‐to‐consumption* model of scaling up, which has seen more success in Bolivia and Peru at the local level (Padulosi et al. 2014). In an effort to bridge the gap between solely academic suggestion and actionable response, useful tools and awareness strategies must be developed for use by decision‐makers. Crop modeling is a powerful tool that enables accurate prediction and planting of appropriate crops and varieties. A major limiting factor for these models is their requirement for many physiological parameters of the crop. Neglected and underutilized crops, by their very nature, lack the required data for these models. A solution to this problem is to use maximum entropy modeling (MaxEnt) as it can make useful geographic predictions about species suitability based on presence‐only data and environmental training data.

MaxEnt has recently been used for local predictions on the suitability of NUS rather than its usual use on ecological species distribution predictions (Koch et al. 2022; Li et al. 2023; Pushpalatha et al. 2023; Reddy et al. 2015). Using current distributions, MaxEnt can predict suitability with future climate data to determine how climate change will affect NUS ranges. Current use of MaxEnt has, understandably, been constrained to individual species range analysis. Given the now widely supported use of MaxEnt on NUS, we seek to use MaxEnt modeling as a case study to create suitability assessments at species‐range scale for use as a simple decision support tool for farmers and decision‐makers worldwide. Our hope is that by combining data cleaning programs and model optimizing programs, we can create a tool framework for decision‐makers for a more confident, data‐driven choice of climate‐resilient crops that will benefit livelihoods now and prepare for changing climate conditions in the future. We will be using previously identified NUS of particular disaster and climate resilience as case studies for this method.

## 2 Methods

### 2.1 Case Study Species Selection

We chose a small set of NUS to investigate based on our previous three literature reviews as a case study. We chose the NUS that were already highlighted in each of the three articles based on their scientific interest, potential for scaling up, nutrition, storability, drought‐resilience, use in disasters, and general resilience (Winstead et al. 2023; Winstead and Jacobson 2022, 2024). This resulted in a list of 44 NUS to be run throughout the MaxEnt modeling process and analysis (Table 3).

### 2.2 Occurrence Data

**FIGURE 1:**
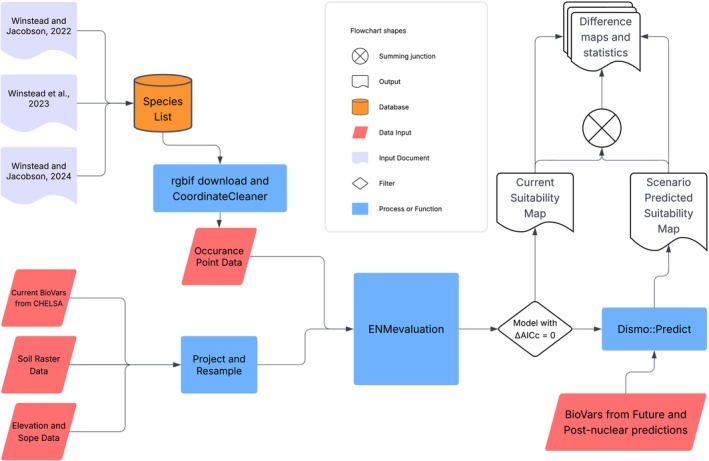
Flowchart of methods.

Occurrence data for these species was collected using the Global Biodiversity Information Facility (GBIF.org) database through the R package “rgbif” with the following criteria: the basis of record must be human observations, living specimens, or occurrence data; likewise, the occurrence point must not have the issue flags of “Country coordinate mismatch,” “zero coordinate,” and “basis of record invalid.” The remaining occurrence points were cleaned using the clean_coordinates() command from the “CoordinateCleaner” R package using the “distance” outlier detection method (Zizka et al. 2019) (Figure 1). Duplicate occurrence points were removed and only one occurrence point was kept per raster cell after raster resampling described below. Occurrence points south of latitude 60°S were omitted.

### 2.3 Training Raster Data

In order to create a parameter system that was consistent enough to possibly be used at various scales in any region of the world, we limited MaxEnt training rasters to the 26 listed in Table 1. A broad set of training rasters was used because our goal was to create a system that allows for the modeling of any NUS that may have completely different predictive variables. This broad list of rasters is further refined for each NUS using the ENMeval::ENMevaluate command described later. For current climate data, we used all 19 bioclimate variables from CHELSA 1981–2010 v.2.1. To accompany climate data, we included soil property data from SoilGrids which were averaged (mean) from 0 to 30 cm depth for Silt, Sand, Clay, and pH data (Khan et al. 2022). Soil depth was retrieved from ORNL DACC (Pelletier et al. 2016). Elevation data was retrieved from WorldClim and slope was calculated from elevation using the terra::terrain function in R.

**TABLE 1:** Training raster variables and descriptions.

Variable name	Description	Unit	Source(s)
BIO1	Annual mean temperature	°C	CHELSA 1981–2010 GFDL‐ESM 4 SSP370 2011–2040 GFDL‐ESM 4 SSP370 2041–2070
BIO2	Mean diurnal range (Mean of monthly (max temp—min temp))	°C	CHELSA 1981–2010 GFDL‐ESM 4 SSP370 2011–2040 GFDL‐ESM 4 SSP370 2041–2070
BIO3	Isothermality (BIO2/BIO7) (×100)		CHELSA 1981–2010 GFDL‐ESM 4 SSP370 2011–2040 GFDL‐ESM 4 SSP370 2041–2070
BIO4	Temperature seasonality (standard deviation ×100)		CHELSA 1981–2010 GFDL‐ESM 4 SSP370 2011–2040 GFDL‐ESM 4 SSP370 2041–2070
BIO5	Max temperature of warmest month	°C	CHELSA 1981–2010 GFDL‐ESM 4 SSP370 2011–2040 GFDL‐ESM 4 SSP370 2041–2070
BIO6	Min temperature of coldest month	°C	CHELSA 1981–2010 GFDL‐ESM 4 SSP370 2011–2040 GFDL‐ESM 4 SSP370 2041–2070
BIO7	Temperature annual range (BIO5–BIO6)	°C	CHELSA 1981–2010 GFDL‐ESM 4 SSP370 2011–2040 GFDL‐ESM 4 SSP370 2041–2070
BIO8	Mean temperature of wettest quarter	°C	CHELSA 1981–2010 GFDL‐ESM 4 SSP370 2011–2040 GFDL‐ESM 4 SSP370 2041–2070
BIO9	Mean temperature of driest quarter	°C	CHELSA 1981–2010 GFDL‐ESM 4 SSP370 2011–2040 GFDL‐ESM 4 SSP370 2041–2070
BIO10	Mean temperature of warmest quarter	°C	CHELSA 1981–2010 GFDL‐ESM 4 SSP370 2011–2040 GFDL‐ESM 4 SSP370 2041–2070
BIO11	Mean temperature of coldest quarter	°C	CHELSA 1981–2010 GFDL‐ESM 4 SSP370 2011–2040 GFDL‐ESM 4 SSP370 2041–2070
BIO12	Annual precipitation	mm	CHELSA 1981–2010 GFDL‐ESM 4 SSP370 2011–2040 GFDL‐ESM 4 SSP370 2041–2070
BIO13	Precipitation of wettest month	mm	CHELSA 1981–2010 GFDL‐ESM 4 SSP370 2011–2040 GFDL‐ESM 4 SSP370 2041–2070
BIO14	Precipitation of driest month	mm	CHELSA 1981–2010 GFDL‐ESM 4 SSP370 2011–2040 GFDL‐ESM 4 SSP370 2041–2070
BIO15	Precipitation seasonality (coefficient of variation)		CHELSA 1981–2010 GFDL‐ESM 4 SSP370 2011–2040 GFDL‐ESM 4 SSP370 2041–2070
BIO16	Precipitation of wettest quarter	mm	CHELSA 1981–2010 GFDL‐ESM 4 SSP370 2011–2040 GFDL‐ESM 4 SSP370 2041–2070
BIO17	Precipitation of driest quarter	mm	CHELSA 1981–2010 GFDL‐ESM 4 SSP370 2011–2040 GFDL‐ESM 4 SSP370 2041–2070
BIO18	Precipitation of warmest quarter	mm	CHELSA 1981–2010 GFDL‐ESM 4 SSP370 2011–2040 GFDL‐ESM 4 SSP370 2041–2070
BIO19	Precipitation of coldest quarter	mm	CHELSA 1981–2010 GFDL‐ESM 4 SSP370 2011–2040 GFDL‐ESM 4 SSP370 2041–2070
Elevation		m	WorldClim
Slope	Calculated from elevation	degrees	
Soil pH		pH	SoilGrids
Soil depth	Soil depth to bedrock	m	ORNL DACC
Clay	Percentage of clay in soil	g/100 g soil	SoilGrids
Silt	Percentage of silt in soil	g/100 g soil	SoilGrids
Sand	Percentage of sand in soil	g/100 g soil	SoilGrids

All training rasters were masked to a buffered area of 500 km around all occurrence points for each species. All predictive outputs were kept to this buffered area to prevent excessive spatial extrapolation. Likewise, we do not intend on using this model to predict crop suitability in areas where it is not currently living. Our intention in this limitation was to prevent suggesting potentially invasive plant introductions.

Bioclimate variable data for future scenarios was retrieved from CHELSA for the 2011–2040 and 2041–2070 GFDL ESM‐4 ssp370 scenario. These bioclimate variables were used to replace the current climate rasters in predictions. All rasters were unprojected to WGS84 if already in a projected coordinate system. Likewise, rasters were resampled to match the resolution of the coarsest variable in this case, the elevation and slope layers.

### 2.4 EMNevaluation

We used the package ENMeval (https://CRAN.R‐project.org/package=ENMeval) to find the optimal MaxEnt model for each NUS in our analysis using an augmented protocol of that described in the ENMeval 2.0 vignette (Kass et al. 2021; Muscarella et al. 2014). Using the command “ENMevaluate” we chose the “k‐fold” spatial validation method (*k* = 10), rm range from 1 to 5 and functions “L, LQ, LQH, H.” If there were too few occurrence points to fulfill the requirements for “H”‐Hinge functions, ENMeval was rerun using only “L, LQ, LQH” functions. These were large, repetitive computations; therefore, they were run in parallel using doParallel on Penn State's ROAR Collab HPC system. The Anaconda environment file containing all required R packages, training rasters, and R script can be found at DOI: https://doi.org/10.5281/zenodo.15528733. For each species’ ENMevaluation, the model with ΔAIC_c_ = 0 was chosen as the most appropriate MaxEnt model to use for that species. If multiple models had ΔAIC_c_ = 0, the model with the highest AUC.test was chosen.

### 2.5 MaxEnt Prediction

The “predict” function from the R package “dismo” was used with the future climate rasters combined with soil, elevation, and slope rasters to predict future suitability for each NUS. We did separate predictions using the 2011–2040 and 2041–2070 data. The resulting predictions were then compared to the original model outputs by subtracting the original model output from the prediction output in each raster cell.

### 2.6 Output and Data Analyses

The output of the R code is designed to produce 15 files (Table 2). Output for each species was normalized using max scaling across each of the three climate scenarios. This was done by dividing all suitability scores by the maximum suitability score found in the three model outputs. This was to ensure that species could not only be compared between climate scenarios within species, but also between species. The statistical analysis file consists of the root mean square (RMS) of differences between current and future climate suitability predictions and the mean change in suitability index from current to future climates.

**TABLE 2:** Workflow output files.

Number	Name	Description	Ext
1	(GBIF download Key)	Occurrence data download from GBIF	.csv
2	(GBIF download Key)_citation	Citation of specific GBIF query with DOI	.txt
3	*_e_mx_soil	ENMeval output	.rds
4	metadata_*	Metadata for ENMevaluation and model output	.csv
5	Pred_*_11–40	ENMeval output for 2011–2040 climate prediction	.rds
6	Pred_*_41–70	ENMeval output for 2041–2070 climate prediction	.rds
7	Suitability_11_40_*	Raster of predicted suitability scores across modeled range for 2011–2040 climate prediction	.tif
8	Suitability_41_70_*	Raster of predicted suitability scores across modeled range for 2041–2070 climate prediction	.tif
9	Suitability_current_*	Raster of suitability values under current climate conditions in modeled range.	.tif
10	Suitability_diff_11_*	Raster of difference in suitability between current conditions and predicted conditions in 2011–2040	.tif
11	Suitability_diff_41_*	Raster of difference in suitability between current conditions and predicted conditions in 2041–2070	.tif
12	SummaryStats_*	calculated summary statistics for species	.csv
13	Suitability_11_40_*_normalized	Normalized values for suitability prediction 2011–2040	.tif
14	Suitability_41_70_*_normalized	Normalized values for suitability prediction 2041–2070	.tif
15	Suitability_current_*_normalized	Normalized values for suitability under current conditions	.tif

* Species name.

## 3 Results and Discussion

**FIGURE 2:**
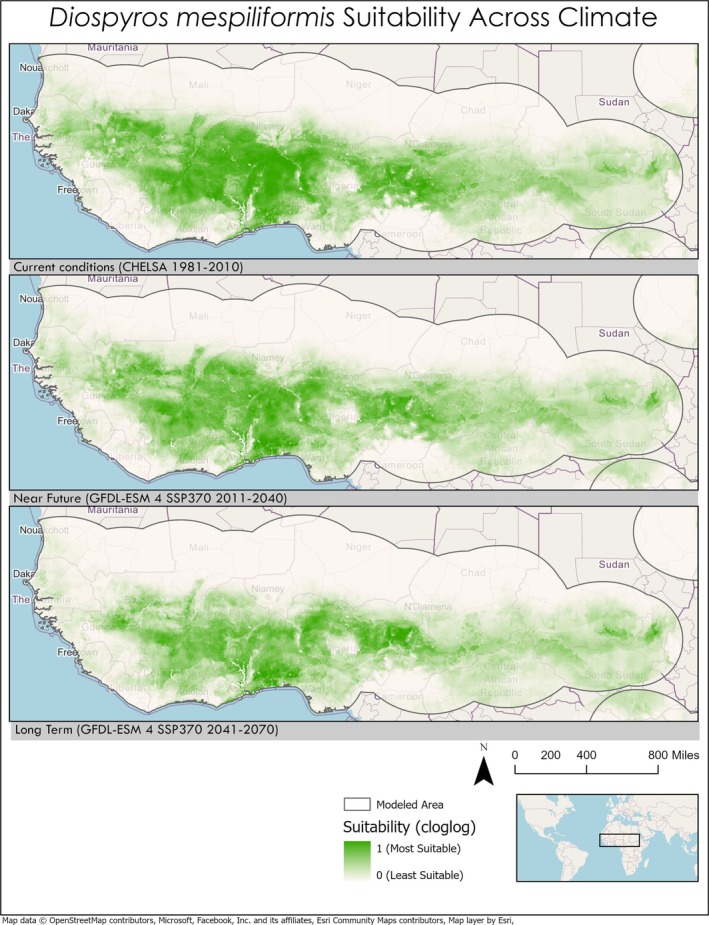
Map of the Sahel region of Africa showing range suitability shifts through time for *Diospyros mespiliformis* . For most of its range, *D. mespiliformis* is predicted to lose suitable area in the future climate model. Map projection: WGS 1984 Web Mercator.

**FIGURE 3:**
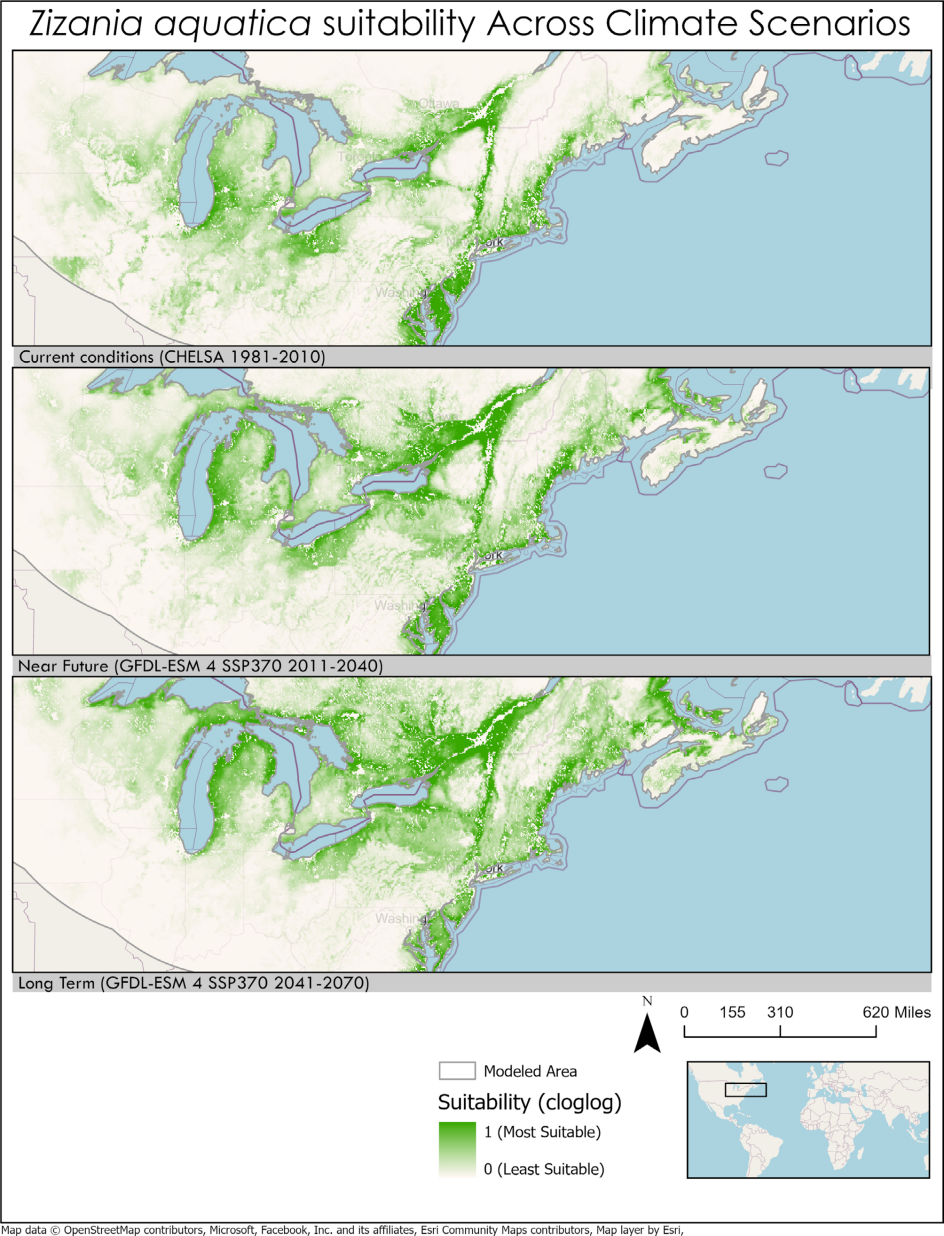
Map of northeast region of North America showing range expansion of *Zizania aquatica* northward. Map projection: WGS 1984 Web Mercator.

**FIGURE 4:**
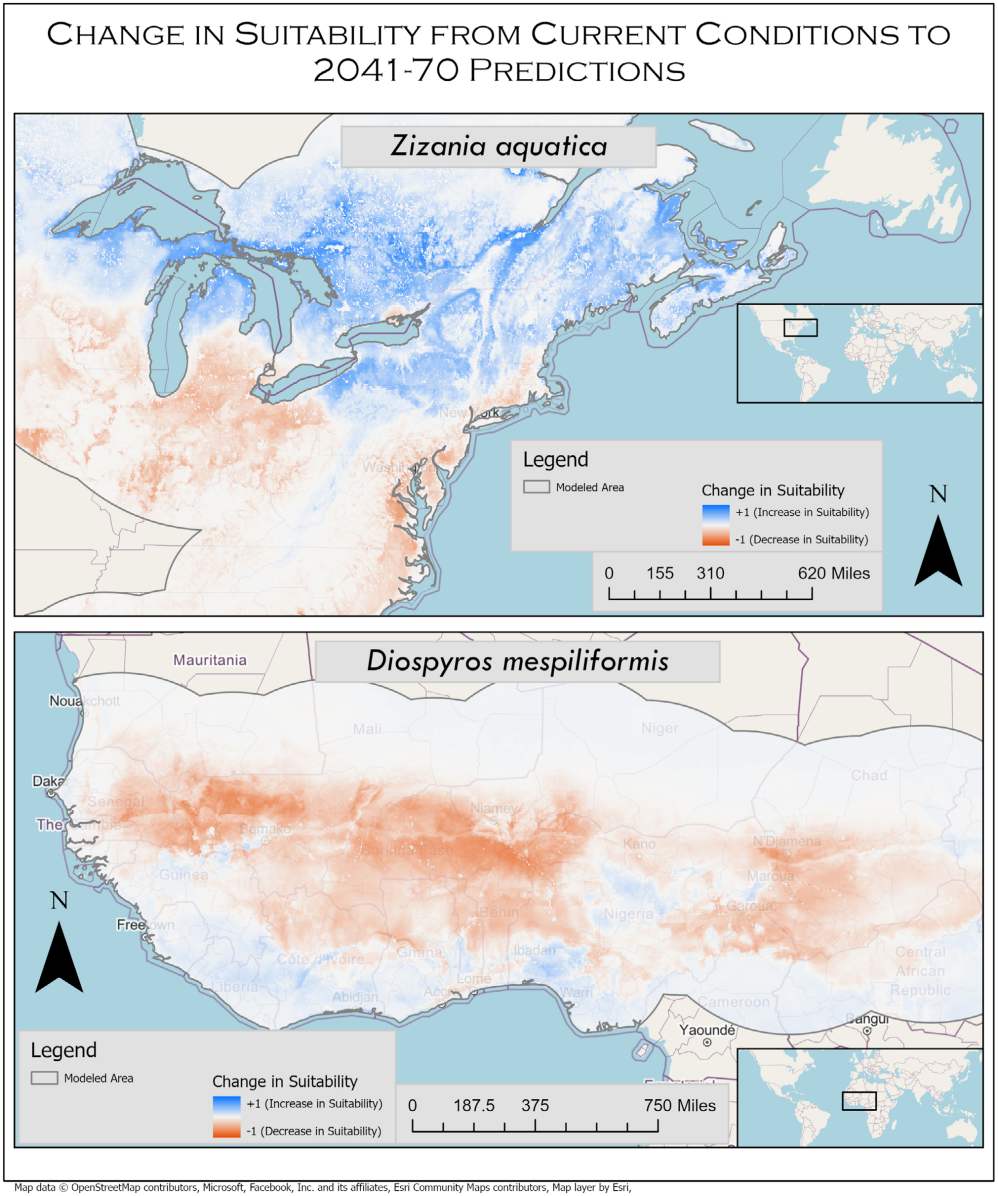
Maps showing change in suitability from current conditions to predicted climates in 2041–2070. Map of *Zizania aquatica* in the northeast portion of North America showing predicted northward range expansion and southern range retraction. Map of *Diospyros mespiliformis* in the Sahel region of Africa depicts a southern expansion of the species toward the coast. Map projection: WGS 1984 Web Mercator.

Changes in suitability from current conditions to predicted climates varied greatly between the NUS chosen in this study (Table 3). Changes in suitability from current conditions to future climate projections are not homogenous across species ranges. For example, *Adansonia digitata* suitability is predicted to increase in eastern Botswana but decrease in most surrounding areas. Similar trends in heterogeneous suitability scores can be seen in other species and regions (Figures 2 and 3). Some species showed little predicted change from current conditions to conditions from GFDL‐ESM 4 SSP370 2041–2070 (e.g., *Carpobrotus edulis* + 0.0007), while others showed large positive or negative changes on average suitability from current conditions across their range (e.g., *Boscia senegalensis* + 0.143, *Plectranthus esculentus* − 0.146). Examples of other species with large changes in suitability include *Zizania aquatica* (+0.092), *Quercus alba* (+0.086), *Amaranthus palmeri* (+0.084), *Diospyros mespiliformis* (−0.063), *Rhodiola rosea* (−0.049), and *Sesbania pachycarpa* (−0.042). Some species saw a shift toward the poles in their range such as *Zizania aquatica* , while others were predicted to decrease throughout their range like *Diospyros mespiliformis* (Figure 4).

**TABLE 3:** Average suitability score difference across climate scenarios.

Species	avg_change_11	avg_change_41	rms_c_11	rms_c_41	Publication	Extant region
*Adansonia digitata*	0.0070	0.0094	0.0459	0.0721	^b^	Af, As
*Agave americana*	−0.0138	−0.0338	0.0450	0.0796	^a^	NA, SA, Af, Eu, As, Oc
*Amaranthus palmeri*	0.0349	0.0840	0.0666	0.1462	^b^	NA, SA, Af, Eu, As, Oc
*Amelanchier alnifolia*	−0.0151	−0.0313	0.0751	0.1275	^a^, ^b^	NA, Eu, As
*Amorphophallus konjac*	−0.0108	−0.0182	0.0411	0.0705	^b^	As
*Arctostaphylos uva‐ursi*	−0.0016	−0.0052	0.0409	0.0689	^a^	NA, Eu, As
*Boscia senegalensis*	0.0829	0.1428	0.1265	0.1985	^a^	Af
*Bromus carinatus*	−0.0101	−0.0112	0.0755	0.1111	^b^	NA, Eu
*Carpobrotus edulis*	0.0028	0.0007	0.0415	0.0703	^c^	NA, SA, Eu, Af, Oc
*Centella asiatica*	−0.0100	−0.0184	0.0373	0.0530	^c^	NA, SA, Af, As, Oc
*Cleome gynandra*	−0.0038	−0.0206	0.0688	0.1259	^c^	NA, SA, Af, As, Oc
*Dacryodes edulis*	−0.0192	−0.0081	0.0613	0.0885	^a^	Af
*Diospyros mespiliformis*	−0.0320	−0.0634	0.0771	0.1333	^c^	Af, As
*Ensete ventricosum*	−0.0075	−0.0198	0.0482	0.0847	^a^	NA, SA, Af
*Gnetum gnemon*	−0.0046	−0.0089	0.0546	0.0577	^a^	Oc
*Helianthus petiolaris*	0.0339	0.0708	0.0601	0.1167	^b^	NA, Eu
*Ipomoea obscura*	−0.0058	−0.0127	0.0499	0.0747	^c^	Af, As, Oc
*Juglans nigra*	0.0328	0.0464	0.0747	0.1233	^b^	NA, Eu
*Lagenaria siceraria*	−0.0088	−0.0123	0.0622	0.1103	^c^	NA, SA, Eu, Af, As, Oc
*Panicum capillare*	0.0044	0.0090	0.0429	0.0744	^c^	NA, Eu, Oc
*Phaseolus acutifolius*	0.0109	0.0201	0.0334	0.0559	^b^	NA
*Physalis pubescens*	0.0224	0.0474	0.0730	0.1335	^b^	NA, SA, Eu, As
*Pinus ponderosa*	−0.0059	−0.0127	0.0392	0.0647	^b^	NA, Eu, Oc
*Plectranthus esculentus*	−0.0804	−0.1461	0.1164	0.2130	^b^	Af
*Prosopis glandulosa*	0.0022	0.0107	0.0356	0.0629	^c^	NA, Af
*Pteridium aquilinum*	0.0051	0.0079	0.0362	0.0678	^b^	NA, SA, Eu, Af, As, Oc
*Quercus alba*	0.0541	0.0864	0.0715	0.1177	^b^	NA, Eu
*Quercus rubra*	0.0325	0.0536	0.0836	0.1563	^b^	NA, Eu
*Rhodiola rosea*	−0.0258	−0.0491	0.0450	0.0768	^b^	NA, Eu, As
*Salvia columbariae*	−0.0033	0.0067	0.0228	0.0345	^b^	NA
*Sclerocarya birrea*	−0.0104	−0.0397	0.0826	0.1321	^b^	Af
*Sesbania pachycarpa*	−0.0101	−0.0420	0.0746	0.1359	^c^	Af
*Strychnos spinosa*	0.0019	−0.0069	0.0270	0.0512	^c^	Af
*Tamarindus indica*	0.0163	0.0263	0.0456	0.0801	^c^	NA, SA, Af, As, Oc
*Typha domingensis*	0.0019	0.0038	0.0403	0.0631	^a^	NA, SA, Eu, Af, As, Oc
*Typha latifolia*	0.0148	0.0271	0.0442	0.0822	^b^	NA, SA, Eu, Af, As, Oc
*Vaccinium myrtillus*	−0.0028	−0.0075	0.0294	0.0476	^b^	NA, Eu, As
*Vangueria infausta*	−0.0112	−0.0197	0.0504	0.0797	^b^	Af
*Xanthium strumarium*	0.0131	0.0190	0.0472	0.0869	^c^	NA, SA, Eu, Af, As, Oc
*Ximenia americana*	−0.0041	−0.0154	0.0557	0.0892	^c^	NA, SA, Af, Oc
*Ximenia caffra*	0.0006	−0.0010	0.0628	0.0852	^c^	Af
*Yucca schidigera*	−0.0135	−0.0145	0.0467	0.0631	^b^	NA
*Zizania aquatica*	0.0560	0.0922	0.1395	0.2195	^b^	NA, Eu, As
*Zizania palustris*	0.0218	0.0447	0.0856	0.1480	^b^	NA, Eu, As

*Note:* Avg_change_11 = average change in suitability score of species across its range from current climate to 2011–2040 climate. Avg_change_41 = same but for 2041–2070 scenario. RMS calculated from all difference values across all raster cells for each comparison. Extant Region determined by GBIF.

Abbreviations: Af, Africa; As, Asia; Eu, Europe; NA, North America; Oc, Oceania; SA, South America.

^a^ Winstead and Jacobson (2022).

^b^ Winstead et al. (2023).

^c^ Winstead and Jacobson (2024).

The results of our initial run of this high‐volume MaxEnt workflow show that some of the NUS highlighted in our previous publications do have increased suitability scores in future climate model projections. Crops that have increased suitability scores in future climate projections should be invested in further as our previous manuscripts have already shown their current interest within academia. By using our MaxEnt workflow we have shown quantitative evidence to support our previous conclusions. However, these models do show that suitability responses by species are not uniform within species' geographies. For instance, many species show areas where suitability scores decrease that are directly adjacent to places where they are shown to have increased suitability (Figure 4). Therefore, decisions should not be made about the use of NUS across large geographic areas but rather determined regionally.

Previous studies have already shown the power of using MaxEnt in predicting crop suitability across time and climate. Our high‐volume MaxEnt workflow shows great promise in its ability to not only show where an NUS is potentially viable, but which NUS may remain viable even in future climate projections. This allows for a sufficient prediction of which NUS to develop by early adopting farmers who are willing to try different crops to combat their current struggles with a changing climate (Ali et al. 2023; Hebbar et al. 2022).

The purpose of using MaxEnt instead of a larger ensemble method is to increase simplicity and usability of this program for those who wish to use it. Likewise, it decreases the amount of computing power necessary to model a species. We understand that the high‐volume automation of MaxEnt lends itself to error. That is a limitation of this design, and any decisions or conclusions made from this model output should be done with great caution. However, we do believe that the large output has value in seeing larger scale trends when comparing many species' reactions to climate change relative to each other, rather than precise single‐species predictions. Our goal is to use these data for the NUS as a starting point for those who may be interested in experimenting with different crops and who are already familiar with the NUS being used.

A good example of the importance of quality checks and further scrutiny of the data is that of *Xanthium strumarium*, which was originally included in our NUS case study list. This species has conclusive research to show that it is poisonous even though it has been cited as a staple food additive by the Costanoan people (Gurley et al. 2010). For this reason, we have excluded it from the results and further discussion.

### 3.1 Future Work in NUS Development

We hope that our NUS suitability tool workflow can function as a bridge between current academic theoretical research on NUS awareness and actual on‐the‐ground development and testing of NUS varieties. In the recent past there has been greater interest by the academic community in using these neglected and underutilized crops, but few actionable steps have been taken by those actually growing crops (Jones et al. 2021). Likewise, future work needs to be done at the local and national policy levels to support a low‐risk environment for smallholder farmers to further develop and test new crop varieties and species (Amanor 2024). We hope that this workflow can be incorporated into an open‐access decision support tool that could be used to help farmers make informed decisions on how to adapt their agriculture in a shifting environment.

## 4 Conclusion

The results from our case study species show the value of this workflow in triaging NUS for future development. We recommend further investigating wild rice (*Zizania* spp.), Palmer amaranth ( *Amaranthus palmeri* ), dilo ( *Boscia senegalensis* ), white oak ( *Quercus alba* ), and prairie sunflower ( *Helianthus petiolaris* ) for further development as resilient crops. These species have already been identified as resilient crop candidates based on their attributes in their respective home ranges. Our models show that these species are likely to expand their range in a “business as usual” future climate scenario. The combination of open‐source species occurrence databases (GBIF), occurrence data cleaning programs (CoordinateCleaner), and automated MaxEnt Model Evaluation (ENMeval) has allowed the creation of a seamless underutilized crop prediction workflow. Our results show that using this workflow could result in the creation of a useful NUS picking tool based on location and future climate.

## Funding

This work was supported by Open Philanthropy Project.

## Conflicts of Interest

The authors declare no conflicts of interest.

## Data Availability

Source code and data inputs are freely available at github.com/danielwinstead/NUSMaxent. Model outputs used in this study are available upon request to the corresponding author.

## References

[pei370115-bib-0001] Ali, S. , T. A. Makanda , M. Umair , and J. Ni . 2023. “MaxEnt Model Strategies to Studying Current and Future Potential Land Suitability Dynamics of Wheat, Soybean and Rice Cultivation Under Climatic Change Scenarios in East Asia.” PLoS One 18: e0296182. 10.1371/journal.pone.0296182. 38127929 PMC10735186

[pei370115-bib-0002] Amanor, K. S. 2024. “Contradictions Between Commercializing Seeds, Empowering Smallholders Farmers, and Promoting Biodiversity in Ghana: Seed Policy Within a Historical Framework.” Elementa, Science of the Anthropocene 12, no. 1: 587–612. 10.1525/elementa.2023.00004.

[pei370115-bib-0003] Belete, T. , and E. Yadete . 2023. “Effect of Mono Cropping on Soil Health and Fertility Management for Sustainable Agriculture Practices: A Review.” Journal of Plant Sciences 11, no. 6: 192–197. 10.11648/j.jps.20231106.13.

[pei370115-bib-0004] Chivenge, P. , T. Mabhaudhi , A. T. Modi , and P. Mafongoya . 2015. “The Potential Role of Neglected and Underutilised Crop Species as Future Crops Under Water Scarce Conditions in Sub‐Saharan Africa.” International Journal of Environmental Research and Public Health 12, no. 6: 5685–5711. 10.3390/ijerph120605685. 26016431 PMC4483666

[pei370115-bib-0005] Gurley, E. S. , M. Rahman , M. J. Hossain , et al. 2010. “Fatal Outbreak From Consuming *Xanthium strumarium* Seedlings During Time of Food Scarcity in Northeastern Bangladesh.” PLoS One 5, no. 3: e9756. 10.1371/journal.pone.0009756. 20305785 PMC2841199

[pei370115-bib-0006] Hebbar, K. B. , P. S. Abhin , V. S. Jose , et al. 2022. “Predicting the Potential Suitable Climate for Coconut (*Cocos nucifera* L.) Cultivation in India Under Climate Change Scenarios Using the MaxEnt Model.” Plants 11, no. 6: 731. 10.3390/plants11060731. 35336613 PMC8954727

[pei370115-bib-0007] Jones, S. K. , N. Estrada‐Carmona , S. D. Juventia , et al. 2021. “Agrobiodiversity Index Scores Show Agrobiodiversity Is Underutilized in National Food Systems.” Nature Food 2, no. 9: 712–723. 10.1038/s43016-021-00344-3. 37117466

[pei370115-bib-0008] Kass, J. M. , R. Muscarella , P. J. Galante , et al. 2021. “ENMeval 2.0: Redesigned for Customizable and Reproducible Modeling of Species' Niches and Distributions.” Methods in Ecology and Evolution 12, no. 9: 1602–1608. 10.1111/2041-210X.13628.

[pei370115-bib-0009] Khan, A. M. , Q. Li , Z. Saqib , et al. 2022. “MaxEnt Modelling and Impact of Climate Change on Habitat Suitability Variations of Economically Important Chilgoza Pine (*Pinus gerardiana* Wall.) in South Asia.” Forests 13, no. 5: 715. 10.3390/f13050715.

[pei370115-bib-0010] Koch, O. , W. A. Mengesha , S. Pironon , et al. 2022. “Modelling Potential Range Expansion of an Underutilised Food Security Crop in Sub‐Saharan Africa.” Environmental Research Letters 17: 014022. 10.1088/1748-9326/ac40b2.

[pei370115-bib-0011] Li, X. , K. Wu , S. Hao , Z. Yue , Z. Ran , and J. Ma . 2023. “Mapping Cropland Suitability in China Using Optimized MaxEnt Model.” Field Crops Research 302: 109064. 10.1016/j.fcr.2023.109064.

[pei370115-bib-0012] Mabhaudhi, T. , V. G. P. Chimonyo , T. P. Chibarabada , and A. T. Modi . 2017. “Developing a Roadmap for Improving Neglected and Underutilized Crops: A Case Study of South Africa.” Frontiers in Plant Science 8: 2143. 10.3389/fpls.2017.02143. 29312397 PMC5735103

[pei370115-bib-0013] Mugiyo, H. , V. G. P. Chimonyo , M. Sibanda , et al. 2021. “Multi‐Criteria Suitability Analysis for Neglected and Underutilised Crop Species in South Africa.” PLoS One 16, no. 1: e0244734. 10.1371/journal.pone.0244734. 33465120 PMC7815157

[pei370115-bib-0014] Muscarella, R. , P. J. Galante , M. Soley‐Guardia , et al. 2014. “ENMeval: An R Package for Conducting Spatially Independent Evaluations and Estimating Optimal Model Complexity for Maxent Ecological Niche Models.” Methods in Ecology and Evolution 5, no. 11: 1198–1205. 10.1111/2041-210X.12261.

[pei370115-bib-0015] National Academies of Sciences, Engineering, and Medicine . 2024. Exploring Linkages Between Soil Health and Human Health. National Academies Press. 10.17226/27459. 39602560

[pei370115-bib-0016] Padulosi, S. , K. Amaya , M. Jäger , E. Gotor , W. Rojas , and R. Valdivia . 2014. “A Holistic Approach to Enhance the Use of Neglected and Underutilized Species: The Case of Andean Grains in Bolivia and Peru.” Sustainability 6, no. 3: 1283–1312. 10.3390/su6031283.

[pei370115-bib-0017] Pelletier, J. D. , P. D. Broxton , P. Hazenberg , et al. 2016. Global 1‐km Gridded Thickness of Soil, Regolith, and Sedimentary Deposit Layers. ORNL DAAC.

[pei370115-bib-0018] Pushpalatha, R. , S. Sunitha , V. Santhosh Mithra , and B. Gangadharan . 2023. “Future Climate Suitability of Underutilized Tropical Tuber Crops‐‘Aroids’ in India.” Journal of Agrometeorology 25, no. 2: 255–261. 10.54386/jam.v25i2.2152.

[pei370115-bib-0019] Reddy, M. T. , H. Begum , N. Sunil , S. R. Pandravada , N. Sivaraj , and S. Kumar . 2015. “Mapping the Climate Suitability Using MaxEnt Modeling Approach for Ceylon Spinach (*Basella alba* L.) Cultivation in India.” Journal of Agricultural Sciences ‐ Sri Lanka 10, no. 2: 87–97. 10.4038/jas.v10i2.8054.

[pei370115-bib-0020] Renwick, L. L. R. , W. Deen , L. Silva , et al. 2021. “Long‐Term Crop Rotation Diversification Enhances Maize Drought Resistance Through Soil Organic Matter.” Environmental Research Letters 16: 084067. 10.1088/1748-9326/ac1468.

[pei370115-bib-0021] Rosero, A. , L. Granda , J. A. Berdugo‐Cely , O. Šamajová , J. Šamaj , and R. Cerkal . 2020. “A Dual Strategy of Breeding for Drought Tolerance and Introducing Drought‐Tolerant, Underutilized Crops Into Production Systems to Enhance Their Resilience to Water Deficiency.” Plants 9: 1263. 10.3390/plants9101263. 32987964 PMC7600178

[pei370115-bib-0022] Tilman, D. , C. Balzer , J. Hill , and B. L. Befort . 2011. “Global Food Demand and the Sustainable Intensification of Agriculture.” Proceedings of the National Academy of Sciences of the United States of America 108, no. 50: 20260–20264. 10.1073/pnas.1116437108. 22106295 PMC3250154

[pei370115-bib-0023] van Dijk, M. , T. Morley , M. L. Rau , and Y. Saghai . 2021. “A Meta‐Analysis of Projected Global Food Demand and Population at Risk of Hunger for the Period 2010–2050.” Nature Food 2, no. 7: 494–501. 10.1038/s43016-021-00322-9. 37117684

[pei370115-bib-0024] Winstead, D. J. , and M. G. Jacobson . 2022. “Food Resilience in a Dark Catastrophe: A New Way of Looking at Tropical Wild Edible Plants.” Ambio 51: 1949–1962. 10.1007/s13280-022-01715-1. 35290618 PMC9287517

[pei370115-bib-0025] Winstead, D. J. , and M. G. Jacobson . 2024. “Storable, Neglected, and Underutilized Species of Southern Africa for Greater Agricultural Resilience.” Plant‐Environment Interactions 5: e70004. 10.1002/pei3.70004. 39183979 PMC11343724

[pei370115-bib-0026] Winstead, D. J. , M. G. Jacobson , and F. Di Gioia . 2023. “Valorizing Staple Native American Food Plants as a Food Resilience Resource.” Frontiers in Sustainable Food Systems 7: 1117805. 10.3389/fsufs.2023.1117805.

[pei370115-bib-0027] Zizka, A. , D. Silvestro , T. Andermann , et al. 2019. “CoordinateCleaner: Standardized Cleaning of Occurrence Records From Biological Collection Databases.” Methods in Ecology and Evolution 10, no. 5: 744–751. 10.1111/2041-210X.13152.

